# Dynamic shifts in the serum lipidome of dogs with obesity before and after weight loss: a pilot study

**DOI:** 10.1093/jas/skag194

**Published:** 2026-06-25

**Authors:** Pedro H Marchi, Leonardo A Príncipe, Daniel S Antonelo, Nicolle P N Gonçalves, Gabriela L F Finardi, Gabriela P T Moreno, Márcia M Jericó, Júlio C C Balieiro, Thiago H A Vendramini

**Affiliations:** Pet Nutrology Research Center (CEPEN Pet), Department of Animal Nutrition and Production, School of Veterinary Medicine and Animal Science, University of Sao Paulo, Pirassununga, SP 13635-900, Brazil; Pet Nutrology Research Center (CEPEN Pet), Department of Animal Nutrition and Production, School of Veterinary Medicine and Animal Science, University of Sao Paulo, Pirassununga, SP 13635-900, Brazil; Lipid Marker Omics Science, Leme, SP 13613-110, Brazil; Pet Nutrology Research Center (CEPEN Pet), Department of Animal Nutrition and Production, School of Veterinary Medicine and Animal Science, University of Sao Paulo, Pirassununga, SP 13635-900, Brazil; Pet Nutrology Research Center (CEPEN Pet), Department of Animal Nutrition and Production, School of Veterinary Medicine and Animal Science, University of Sao Paulo, Pirassununga, SP 13635-900, Brazil; Veterinary Nutrology Service, Veterinary Teaching Hospital, School of Veterinary Medicine and Animal Science, University of Sao Paulo, Sao Paulo, SP 05508-270, Brazil; Alto da Lapa Veterinary Clinics, MV Minds Clinical Research and Continuing Education, São Paulo, SP 05415-011, Brazil; Pet Nutrology Research Center (CEPEN Pet), Department of Animal Nutrition and Production, School of Veterinary Medicine and Animal Science, University of Sao Paulo, Pirassununga, SP 13635-900, Brazil; Pet Nutrology Research Center (CEPEN Pet), Department of Animal Nutrition and Production, School of Veterinary Medicine and Animal Science, University of Sao Paulo, Pirassununga, SP 13635-900, Brazil; Veterinary Nutrology Service, Veterinary Teaching Hospital, School of Veterinary Medicine and Animal Science, University of Sao Paulo, Sao Paulo, SP 05508-270, Brazil

**Keywords:** canine, lipid metabolism, obesity, omics sciences, weight reduction

## Abstract

Canine obesity is associated with metabolic disturbances, but specific lipid alterations remain poorly characterized. This study aimed to characterize the serum lipidome of dogs with obesity before and after weight loss (WL) and to identify lipid species and pathways associated with lipidomic remodeling following weight loss. Eight neutered dogs with obesity (four males and four females; 5.37 ± 1.41 years; 27.15 ± 12.02 kg; body condition score 9/9) were fed a commercial weight-loss diet. After a 3-wk adaptation, blood was collected for lipidomic analysis before weight loss (OB). A weight-loss program was implemented for up to 6 months until dogs achieved a 20% reduction from initial body weight, followed by a second blood collection after weight loss. Targeted lipidomic profiling was performed using two multiple reaction monitoring (MRM) methods on a triple quadrupole mass spectrometer, covering 3,437 lipid ion transitions. Data were processed in MetaboAnalyst 6.0 and analyzed using Student’s t-test, volcano plot, partial least squares-discriminant analysis (PLS-DA), variable importance in projection (VIP) scoring, and Lipid Ontology (LION) enrichment analysis. Dogs achieved a total weight loss of 23.68 ± 3.18%, with a weekly rate of 1.01 ± 0.26%. A total of 630 lipid species were identified, and clear remodeling of the canine serum lipidome was observed following weight loss. Before weight loss, dogs had higher serum concentrations of cholesterol esters (*P *= 0.047), phosphatidylcholines (*P *= 0.043), phosphatidylinositols (*P *= 0.038), and sphingomyelins (*P *= 0.023) than after weight loss. At the individual lipid-species level, volcano plot analysis identified 34 differentially abundant lipid species before and after weight loss, including specific triglyceride, phospholipid, and cholesterol ester species. PLS-DA clearly discriminated the serum lipidomic profiles before and after weight loss with high predictive performance. Additionally, LION enrichment analysis revealed multiple significantly enriched terms related to lipid cellular components, functional roles, and physicochemical properties. These findings provide novel insights into serum lipidomic remodeling associated with weight loss in dogs with obesity and highlight lipid species potentially involved in the metabolic response to weight reduction.

## Teaser

This study provides the first serum lipidomic characterization of dogs with obesity before and after weight loss, revealing metabolic alterations not detected by conventional lipid markers. By uncovering dynamic lipid remodeling after weight reduction, these findings highlight novel lipid pathways and metabolic adaptations associated with weight loss in dogs with obesity.

## Introduction

Obesity leads to a complex network of altered physiological responses in dogs. Excessive adipose expansion is associated with chronic low-grade inflammation, insulin resistance, altered adipokine secretion, and hyperlipidemia, which contribute to obesity-related comorbidities (German et al. 2009; Brunetto et al. 2011; Tvarijonaviciute et al. 2012). The mechanisms underlying the development of such conditions often overlap, which makes it difficult to establish clear cause-and-effect relationships (Marchi et al. 2022). In the context of current scientific understanding of lipid metabolism in dogs with obesity, a significant focus is directed toward hypertriglyceridemia, hypercholesterolemia, and imbalances in lipoprotein levels (Mori et al. 2011; Gomez-Fernandez-Blanco et al. 2024). On the other hand, several other lipid classes and species have already been established as harmful or at least correlated with obesity in humans (Torretta et al. 2019).

Studies applying serum metabolomics in dogs with obesity have reported alterations in lipid synthesis and metabolism, including increased concentrations of lipoproteins, phospholipids, and phosphatidylcholines, as well as reduced levels of taurine and carnitine (Söder et al. 2017, 2019; Forster et al. 2018; Vendramini et al. 2021; Qu et al. 2022; Cai et al. 2024). However, although metabolomics identifies certain lipid classes, its analyses are limited due to the physicochemical particularities of these molecules (Domenick et al. 2021; Han and Gross 2022). In this sense, lipidomics emerged as a tool for targeted investigation of thousands of lipid species, highlighting precise alterations in the lipid profile (Han 2016).

The advancements catalyzed by the advent of lipidomics and by the growing understanding of the role of lipids in the development and progression of obesity and other human diseases are evident (Yang et al. 2016). However, the pathophysiological basis of obesity in dogs linking altered lipid metabolism to metabolic dysfunction and to metabolic improvement during weight loss remains largely unknown. By enabling a detailed and pathway-oriented characterization of lipid metabolism, serum lipidomics represents a logical next step to advance the understanding of obesity-related metabolic dysfunction in dogs. Therefore, the present study aimed to characterize the serum lipidome of dogs with obesity before and after a controlled weight-loss program and to evaluate lipidomic remodeling associated with a reduction of at least 20% of initial body weight.

## Materials and methods

All procedures performed in this study were approved by the Ethics Committee on Animal Use of the School of Veterinary Medicine and Animal Science, University of Sao Paulo (CEUA/FMVZ), under Protocol Number 4668091214. All owners provided informed consent for their dogs to participate in the study. Owner participation was limited to providing consent, following home feeding management instructions, and attending follow-up appointments; therefore, no separate human research ethics approval was required.

### Experimental design

Client-owned dogs with preclinical obesity were recruited through electronic advertisement and evaluated at the Pet Nutrology Research Center (CEPEN Pet), School of Veterinary Medicine and Animal Science, University of Sao Paulo, Pirassununga, SP, Brazil. Preclinical obesity was defined as excessive adiposity without major signs of obesity-related organ dysfunction or limitations in daily living activities (German et al. 2025). Eligible dogs were neutered males or spayed females, 1 to 8 yr of age, with a body condition score (BCS) ≥ 8 on a 9-point scale (Laflamme 1997), and with physical examination and laboratory results within the reference intervals for the species.

To minimize potential confounding effects on the serum lipidome, dogs with hypercholesterolemia (serum cholesterol > 270.0 mg/dL), hypertriglyceridemia (serum triglycerides > 169.0 mg/dL), cardiorespiratory, locomotor, hepatic, gastrointestinal, renal, endocrine, or other concomitant disorders were not included. Dogs that had received pharmacological treatment within the 6 wk preceding the study were also excluded. In addition, owners had to agree to follow the proposed feeding management and experimental protocol. Dogs remained housed in their owners’ homes throughout the study. Therefore, housing conditions varied according to each household environment, including houses or apartments. During the weight-loss program, dogs maintained their habitual owner-managed physical activity routine, while owners were encouraged to promote regular activity, such as daily walks.

The animals consumed the same commercial weight-loss diet formulated for dogs with obesity (Pro Plan OM Overweight Management, Nestlé Purina, Brazil) throughout the entire experiment, including both the 3-wk adaptation period and the weight-loss protocol. Owners were instructed not to provide treats, table scraps, or any additional foods throughout the entire experiment. During the adaptation period, the diet was standardized and the maintenance energy requirement was determined while dogs consumed this diet. The maintenance energy requirement (MER) was calculated as $\mathrm{MER}\;(\frac{\mathrm{kcal}}{\mathrm{day}})=95\times {\mathrm{current}\;\mathrm{body}\;\mathrm{weight}\;(\mathrm{kg})}^{0.75}$, according to FEDIAF (2025). After this period, blood was collected using serum separator tubes (yellow-top tubes) to obtain serum for lipidomic analysis before weight loss (OB). Dogs then began the weight-loss protocol.

The weight-loss energy requirement (WLER) was calculated based on target body weight (TW) using the equation: $\mathrm{WLER}\;(\frac{\mathrm{kcal}}{\mathrm{day}})=70\;\times {\mathrm{TW}}^{0.75}$, where TW was defined as the initial body weight minus 20%. This magnitude of weight loss was selected based on previous studies in dogs with obesity, in which a 20% reduction in body weight was associated with changes in glucose metabolism, respiratory function, and serum metabolomic profiles, while allowing a standardized experimental approach (Brunetto et al. 2011; Pereira-Neto et al. 2018; Vendramini et al. 2021). Based on this equation, the animals initially consumed between 60% and 70% of their MER.

During the weight-loss protocol, the same diet was maintained, and only the amount of food offered was adjusted according to each dog’s weight-loss response. Dogs were re-evaluated every 15 to 21 d, according to the owners’ availability to attend follow-up appointments, for body weight, BCS, and muscle mass score (MMS) assessment. The weekly weight-loss rate was calculated as follows: $(\frac{\mathrm{previous}\;\mathrm{body}\;\mathrm{weight}-\mathrm{current}\;\mathrm{body}\;\mathrm{weight}}{\mathrm{previous}\;\mathrm{body}\;\mathrm{weight}}\times 100)÷\mathrm{number}\;\mathrm{of}\mathrm{weeks}.$ The expected weekly weight-loss rate was 1% to 2%, according to the American Animal Hospital Association Weight Management Guidelines for Dogs and Cats (Brooks et al. 2014). Energy intake was adjusted by approximately 10% when necessary to maintain the desired rate of weight loss.

Dogs were maintained on the weight-loss protocol until achieving at least 20% reduction from initial body weight and a BCS of 4–5 out of 9. Therefore, the 20% reduction was considered the minimum weight-loss target, whereas a BCS of 4–5 out of 9 was used as the clinical endpoint and safety threshold to avoid excessive weight loss. Dogs whose weight-loss process exceeded six months were excluded from the study. At the end of the weight-loss protocol, blood was collected again from the same dogs for serum lipidomic analysis after WL.

### Chemical analysis of the diet

Dry matter and ash content were determined according to the methods described by AOAC (2006). Crude fiber was determined by sequential digestion of the sample with 1.25% sulfuric acid and sodium hydroxide in a Soxhlet extractor, followed by drying and ashing (AOAC 2006; method 962.09). Ether extract was determined by petroleum ether extraction in a Soxhlet apparatus (AOAC 2006; method 942.05). Crude protein was determined using the Micro-Kjeldahl method, with the total nitrogen value multiplied by 6.25 (AOAC 2006; method 954.01). Organic matter was calculated by 100 – ash (%), and the nitrogen-free extracts were calculated by 100 – (crude protein + ether extract + crude fiber + ash). Gross energy was measured using an oxygen bomb calorimeter (model C–5003, IKA-Werke GmbH & CO. KG, Staufen, Germany). Metabolizable energy was calculated using the equation proposed by the NRC (National Research Council) (2006), and the information provided on the label was also considered.

### Complete blood count and biochemical profile

Health status was assessed through physical examination, complete blood count, and biochemical analyses, including urea, creatinine, alanine aminotransferase, alkaline phosphatase, total proteins, albumin, globulins, total cholesterol, triglycerides, and glucose. Blood samples were collected after a 12-h fasting period. A total of 5 mL of whole blood was collected from each dog and divided as follows: 0.5 mL into EDTA tubes (BD Vacutainer; Becton, Dickinson, and Co., Franklin Lakes, NJ) for complete blood count, 0.5 mL into fluoride tubes (BD Vacutainer; Becton, Dickinson, and Co.) for glucose measurement, and 4 mL into serum separator tubes with clot activator (yellow-top tubes; BD Vacutainer; Becton, Dickinson, and Co.) for serum biochemical analyses. Whole blood collected in serum separator tubes was centrifuged at 3,500 rpm for 15 min at 4 °C to obtain serum for subsequent biochemical analyses.

Serum biochemical concentrations were determined using an automated spectrophotometer Mindray BS 120 (Mindray, Shenzhen Mindray Bio-Medical Electronics Co., Ltd., Shenzhen, China) and commercial kits from Labtest Diagnóstica S.A. (Lagoa Santa, MG, Brazil): urea (Ref. 104–4), creatinine (Ref. 96), alanine aminotransferase (Ref. 108–4/30), alkaline phosphatase (Ref. 79–4/30), total proteins (Ref. 99–100), albumin (Ref. 19/250), triglycerides (Ref. 87–2/100), total cholesterol (Ref. 76–2/100), and glucose (Ref. 133–2/500). Calibra H (Ref. 80–1) and Qualitrol 1H (Ref. 71–1) were used for calibration and quality control, respectively. Globulin concentrations were calculated mathematically. Hematological analyses were performed using the automated hematology analyzer Mindray BC-2800 Vet (Mindray, Shenzhen Mindray Bio-Medical Electronics Co., Ltd., Shenzhen, China).

### Lipidome analysis

#### Lipid extraction

A 200 µL aliquot of each serum sample collected before and after weight loss was used for lipid extraction using the method reported by Bligh and Dyer (1959). Briefly, 300 μL of chloroform and 300 μL of methanol were added to samples and vigorously vortexed for 10 s. The solution was incubated at 4 °C for 10 min and centrifuged at 10,000×*g* for 10 min at 4 °C. The lower organic phase containing lipids was transferred to separate vials and evaporated to dryness in a SpeedVac concentrator (Genevac™ miVac, Genevac Ltd., Ipswich, UK). The dried lipid extracts were stored at −80°C until further analysis.

#### Multiple reaction monitoring-profiling

Targeted lipid profiling was performed using discovery multiple reaction monitoring (MRM)-profiling methods and instrumentation as reviewed by Xie et al. (2021). Specifically, dried lipid extracts were diluted in 50 μL of methanol/chloroform 3:1 (v/v) and 300 μL of injection solvent (acetonitrile/methanol/300 mM ammonium acetate 3:6.65:0.35 [v/v/v]) to obtain a stock solution. Mass spectrometry data were acquired by flow-injection (no chromatographic separation) from 15 μL of stock solution that was diluted 150-fold in injection solvent and spiked with EquiSPLASH™ LIPIDOMIX Quantitative Mass Spec Internal Standard (0.1 ng/µL) prior to being delivered to the electrospray ionization source of an Agilent 6410 triple quadrupole mass spectrometer (Agilent Technologies, Santa Clara, CA, USA) using a micro-autosampler (G1377A). A capillary pump was coupled to the autosampler and operated at a flow rate of 10 μL/min and pressure of 150 bar. The capillary voltage of the instrument was 4 kV, and the gas flow 5.1 L/min at 300 °C.

The MRM profiling method was used to profile 3,437 MRMs related to lipids, from 12 lipid classes. The MRM set included 1,752 triglycerides (TG), 482 diglycerides (DG), 179 free fatty acids (FFA), 178 phosphatidylcholines (PC), 145 phosphatidylethanolamines (PE), 141 phosphatidylinositols (PI), 141 phosphatidylglycerols (PG), 139 phosphatidylserines (PS), 120 ceramides (CER), 84 acyl carnitines (CAR), 42 sphingomyelins (SM), and 34 cholesterol esters (CE). TG were profiled using parent ions and a product ion related to the presence of specific fatty acyl residues. Ion intensity data of each MRM per sample was obtained using in-house scripts. To minimize MRM noise, only ion intensities greater than 1.3-fold the ion intensity of blanks were considered as a valid MRM. Relative ion intensities were calculated for each valid MRM by dividing its ion intensity by the sum of all ion intensities across each sample. To reduce overlaps and increase the analytical potential of each lipid class, TG and DG, which have the highest numbers of profiled lipids (2,234 out of 3,437 MRMs), were analyzed in a separate method from the other lipid classes. Therefore, MRM were then assigned to one of two lipid methods: (1) TG and DG; (2) CAR, CE, CER, PC, PE, PG, PI, PS, and SM, which were used for subsequent statistical analysis and bioinformatics. Detailed information on lipid extraction and MRM-profiling methods can be found in Antonelo et al. (2022).

### Statistical analysis and bioinformatics

Lipidome data were uploaded to MetaboAnalyst 6.0 (https://www.metaboanalyst.ca/) (Chong et al. 2019) and data were auto-scaled prior to statistical and bioinformatics analyses. Differences in TG and DG distribution before and after weight loss were analyzed using Student’s t-test according to three distinct groupings: total number of carbons (e.g. TG(54) or DG(40)); number of unsaturations; and unsaturation class, with TG and DG grouped as monounsaturated (up to two unsaturations) or polyunsaturated (more than three unsaturations). The internal standard was used to obtain the relative quantification of each lipid class. Differences were considered statistically significant when *P *≤ 0.05.

Volcano plot analysis (*P *≤ 0.05; Fold change ≥ 1.5) was conducted to investigate the effects of weight loss on serum lipidome profile. Partial least squares-discriminant analysis (PLS-DA) was performed. PLS-DA validation was performed using a 10-fold cross validation method, and the values for *R*^2^ (cumulative interpretation ability of the model), Q^2^ (predictive ability of the model) and accuracy were employed as initial indicators for evaluating the goodness of fit. In the PLS-DA model, a variable importance in projection (VIP) plot was used to rank the lipids based on their relative importance of variation in discriminating groups.

In addition, lipidome data were further processed for Lipid Ontology (LION) enrichment analysis (https://www.lipidontology.com) using lipid quantification data sets before and after weight loss, according to Molenaar et al. (2019). The compound name was standardized according to LIPID MAPS Structure Database (https://lipidmaps.org/) and functional analysis was performed by preselecting cellular component, function, lipid classification and physical or chemical properties as ontology sources in the ranking mode. LION-terms were mapped based on *P*value corrected for multiple testing using the false discovery rate (FDR ≤ 0.05).

## Results

### Study population and weight-loss outcome

A total of 13 client-owned dogs were screened for eligibility. Five dogs did not complete the study: three were withdrawn due to owner discontinuation, and two were excluded because they did not achieve the target weight loss within 6 months. Therefore, eight client-owned dogs with obesity completed the study, including four neutered males and four spayed females, with a mean age of 5.37 ± 1.41 years (range: 3–8 years) and body weight of 27.15 ± 12.02 kg (range: 5.3–38.4 kg; Table 1). Dogs had a BCS of 9 ± 0 out of 9 (Laflamme 1997) and MMS of 2.87 ± 0.3 out of 3 (Michel et al. 2011). The study population included Miniature Pinscher (*n* = 1), mixed-breed dogs (*n* = 2), Labrador Retriever (*n *= 1), Dalmatian (*n *= 1), Australian Cattle Dog (*n* = 2), and Dachshund (*n* = 1).

**Table 1:** Individual traits and weight-loss outcomes of the animals included in the study.

Variable	Dog 1	Dog 2	Dog 3	Dog 4	Dog 5	Dog 6	Dog 7	Dog 8
**Age, yr**	5	5	8	3	6	6	5	5
**Breed**	Miniature Pinscher	Mixed Breed	Labrador Retriever	Dalmatian	Mixed Breed	Australian Cattle Dog	Australian Cattle Dog	Dachshund
**Sex**	Male	Female	Male	Female	Female	Male	Female	Male
**Before weight loss**								
** Body weight, kg**	5.3	34.5	38.0	38.4	24.3	36.5	24.6	15.6
** Food intake to maintain weight, g**	99	404	435	438	347	422	314	223
** Energy intake per kg of metabolic body weight, kcal**	84.7	84.9	85.0	84.9	94.8	85.0	85.0	84.9
** Fat intake per kg of metabolic body weight, g**	1.61	1.62	1.61	1.62	1.80	1.61	1.62	1.67
** BCS^1^**	9	9	9	9	9	9	9	9
** MMS** ^2^	3	3	2	3	3	3	3	3
**After weight loss**								
** Body weight, kg**	4.1	26.5	30.0	30.1	19.1	26.8	18.7	10.4
** Food intake to reduce weight, g**	38	266	203	146	142	184	197	122
** Energy intake per kg of metabolic body weight, kcal**	39.4	68.1	47.4	34.0	46.5	46.7	65.5	63.0
** Fat intake per kg of metabolic body weight, g**	0.73	1.29	0.90	0.64	0.88	0.89	1.24	1.20
** BCS^1^**	5	4	5	5	5	4	5	4
** MMS^2^**	3	3	2	3	3	3	3	3
** Average weekly weight loss, %**	1.0	1.46	0.88	0.64	0.74	1.03	1.21	1.12
** Total weight loss, %**	22.6	22.5	21.05	21.4	21.4	26.6	23.7	30.2
** Weeks to achieve total weight loss**	23.0	14.4	22.7	23.7	23.4	22.4	19.3	23.0

1 Body condition score based on Laflamme (1997).

2 Muscle mass score based on Michel et al. (2011).

The weight-loss program effectively reduced body weight and improved BCS in all eight dogs with obesity (Table 1). Dogs initially weighed 27.15 ± 12.02 kg, ranging from 5.3 to 38.4 kg, and all started with a BCS of 9 out of 9. By the end of the program, body weight was reduced to 20.71 ± 9.52 kg, ranging from 4.1 to 30.1 kg, and BCS improved to 4.63 ± 0.52, ranging from 4 to 5 out of 9. Weekly weight loss averaged 1.01 ± 0.26%, ranging from 0.64% to 1.46%, with a total reduction of 23.68 ± 3.18%, ranging from 21.05% to 30.2%. Food intake was adjusted according to the WLER, resulting in a reduced daily intake for all dogs. The program successfully achieved the target weight loss with no reported adverse health effects.

### Diet composition analysis

The results of the chemical composition analysis of the diet used in the study are described in Table 2. The analyzed nutrient profile was consistent with the values declared by the manufacturer for the Brazilian market. As expected for a commercial diet formulated for weight management, the diet was characterized by a high crude protein content and reduced lipid concentration and caloric density.

**Table 2:** Chemical composition of the commercial diet used in the study.

Variable	Obesity diet^1^
**Dry matter, %**	92.27
	Dry matter basis
**Organic matter, %**	92.19
**Crude protein, %**	30.43
**Fat, %**	5.68
**Crude fiber, %**	4.46
**Ash, %**	7.81
**Nitrogen-free extract, %^2^**	51.62
**Calcium, %**	1.19
**Phosphorus, %**	0.85
**Gross energy, kcal/g^3^**	4.278
**Calculated ME, kcal/g^4^**	3.312
**Label ME, kcal/g^5^**	2.990

ME, metabolizable energy.

1 Pro Plan OM Overweight Management, Nestlé Purina, Brazil. Ingredients: ground whole corn, corn gluten meal - 60, soybean fiber (11%), poultry by-product meal, soybean germ meal (source of isoflavones), soybean meal, wheat gluten, fish meal, powdered cellulose (2%), beef tallow, dicalcium phosphate, sodium chloride (common salt), potassium chloride, hydrolyzed poultry by-products, DL-methionine, L-lysine, vitamins (A, D3, E, K3, B1, B2, niacin, pantothenic acid, B6, biotin, folic acid, B12, choline chloride), minerals (zinc sulfate, iron sulfate, manganese sulfate, copper sulfate, zinc proteinate, iron proteinate, manganese proteinate, copper proteinate, selenium proteinate, calcium iodate, sodium selenite), and antioxidant (BHT). Potential substitutes: hydrolyzed chicken liver, hydrolyzed pork liver, calcium carbonate.

2 Nitrogen-free extract = 100 − (crude protein + fat + crude fiber + ash).

3 Gross energy measured by calorimetry.

4 Metabolizable energy calculated according to Kienzle et al. (1998) and NRC (National Research Council) (2006):

Energy digestibility, % = 91.2 – (1.43 × % crude fiber)

Digestible energy, kcal = (gross energy × energy digestibility)/100

Metabolizable energy, kcal = digestible energy – (1.04 × % crude protein).

5 Information declared on the label.

### Lipidome analysis

Out of the 3,437 ion transitions (MRMs) scanned in a pooled sample for identifying the detectable lipids, 630 (*n* = 363 in method 1 [Table S1]; and *n* = 267 in method 2 [Table S2]) had intensities of at least 1.3-fold higher than the blank and were used for interrogating individual samples. These compounds were related mainly to TG (301), PC (119), DG (62), SM (38), and CE (37) lipids.

**Figure 1:**
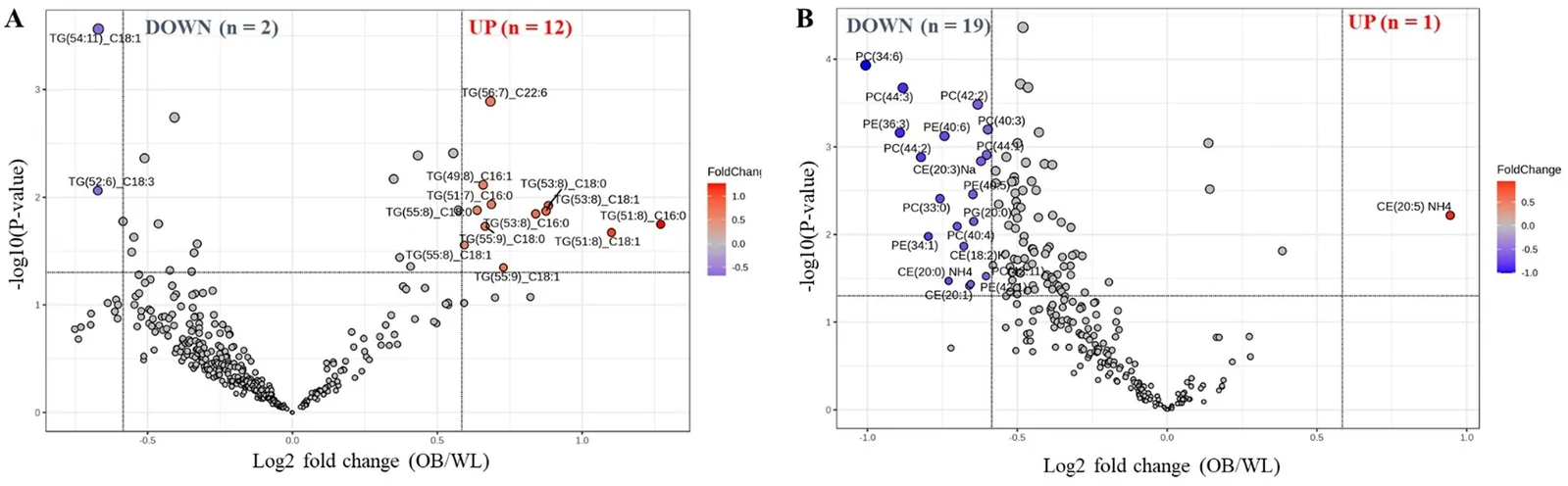
Volcano plot analysis showing the serum lipids that significantly differed (fold change ≥ 1.5 and *P*value ≤ 0.05) in method 1 (A; triglyceride [TG] and diglyceride [DG]; *n* = 363) and method 2 (B; acyl-carnitine [CAR], cholesterol ester [CE], ceramides [CER], phosphatidylcholine [PC], phosphatidylethanolamine [PE], phosphatidylglycerol [PG], phosphatidylinositol [PI] and phosphatidylserine [PS], and sphingomyelin [SM]; *n* = 267) in dogs with obesity before (OB) and after weight loss (WL). Lipids up-regulated in OB compared with WL are shown on the right side of the plot and indicated in red, while lipids down-regulated in OB compared with WL are shown on the left side of the plot and indicated in blue.

Volcano plot analyses allowed identifying differentially abundant lipids (DAL) before and after weight loss (Figure 1; Table 3). From the 14 DAL identified in method 1, 12 were up-regulated and two were down-regulated in OB compared with WL (Figure 1A), whereas from the 20 DAL identified in method 2, one was up-regulated and 19 were down-regulated in OB compared with WL (Figure 1B).

**Table 3:** Differentially abundant lipids identified in dogs with obesity before and after weight loss.

	Up-regulated in	FC	Log2(FC)	*P-*value	−Log10(*P*)
**Method 1**					
** TG(54:11)_C18:1**	WL	0.63	−0.67	<0.001	3.562
** TG(56:7)_C22:6**	OB	1.61	0.68	0.001	2.891
** TG(49:8)_C16:1**	OB	1.58	0.66	0.008	2.114
** TG(52:6)_C18:3**	WL	0.63	−0.67	0.009	2.061
** TG(51:7)_C16:0**	OB	1.61	0.69	0.012	1.934
** TG(53:8)_C18:0**	OB	1.85	0.88	0.012	1.923
** TG(55:8)_C18:0**	OB	1.56	0.64	0.013	1.879
** TG(53:8)_C18:1**	OB	1.83	0.88	0.013	1.872
** TG(53:8)_C16:0**	OB	1.79	0.84	0.014	1.845
** TG(51:8)_C16:0**	OB	2.41	1.27	0.018	1.748
** TG(55:9)_C18:0**	OB	1.59	0.67	0.019	1.730
** TG(51:8)_C18:1**	OB	2.15	1.10	0.021	1.672
** TG(55:8)_C18:1**	OB	1.51	0.59	0.028	1.556
** TG(55:9)_C18:1**	OB	1.66	0.73	0.045	1.347
**Method 2**					
** PC(34:6)**	WL	0.50	−1.01	<0.001	3.932
** PC(44:3)**	WL	0.54	−0.88	<0.001	3.672
** PC(42:2)**	WL	0.65	−0.63	<0.001	3.483
** PC(40:3)**	WL	0.66	−0.60	<0.001	3.198
** PE(36:3)**	WL	0.54	−0.89	<0.001	3.160
** PE(40:6)**	WL	0.60	−0.74	<0.001	3.123
** PC(44:1)**	WL	0.66	−0.60	0.001	2.908
** PC(44:2)**	WL	0.57	−0.82	0.001	2.882
** CE(20:3) Na**	WL	0.65	−0.62	0.001	2.837
** PE(40:5)**	WL	0.64	−0.65	0.003	2.457
** PC(33:0)**	WL	0.59	−0.76	0.004	2.409
** CE(20:5) NH4**	OB	1.92	0.94	0.006	2.218
** PG(20:0)**	WL	0.64	−0.65	0.007	2.149
** PC(40:4)**	WL	0.62	−0.70	0.008	2.093
** PE(34:1)**	WL	0.58	−0.80	0.011	1.978
** CE(18:2) K**	WL	0.62	−0.68	0.014	1.867
** PC(42:11)**	WL	0.66	−0.60	0.030	1.524
** CE(20:0) NH4**	WL	0.60	−0.73	0.034	1.470
** PE(42:1)**	WL	0.63	−0.66	0.037	1.435
** CE(20:1)**	WL	0.63	−0.66	0.039	1.415

CE, cholesterol ester; FC, fold change; OB, dogs with obesity before weight loss; PC, phosphatidylcholine; PE, phosphatidylethanolamine; PG, phosphatidylglycerol; TG, triglyceride; WL, dogs after weight loss. Differentially abundant lipids were identified using fold change ≥ 1.5 and *P-*value ≤ 0.05.

**Figure 2:**
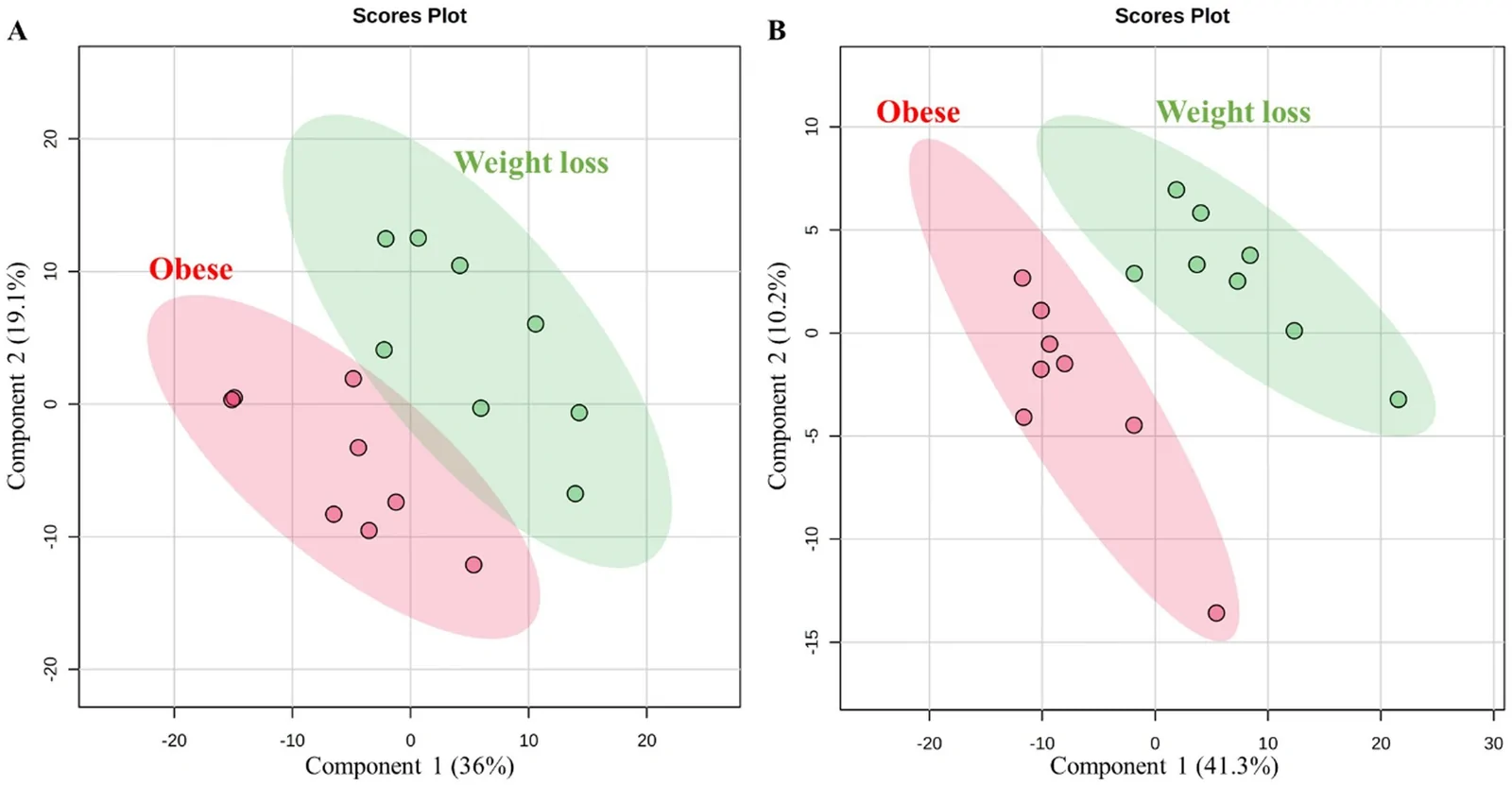
Partial least squares-discriminant analysis (PLS-DA) plot of serum lipids distribution in method 1 (A; triglyceride and diglyceride; *n* = 363) and method 2 (B; acyl-carnitine, cholesterol ester, ceramides, phosphatidylcholine, phosphatidylethanolamine, phosphatidylglycerol, phosphatidylinositol and phosphatidylserine, and sphingomyelin; *n* = 267) in dogs with obesity before and after weight loss.

**Figure 3:**
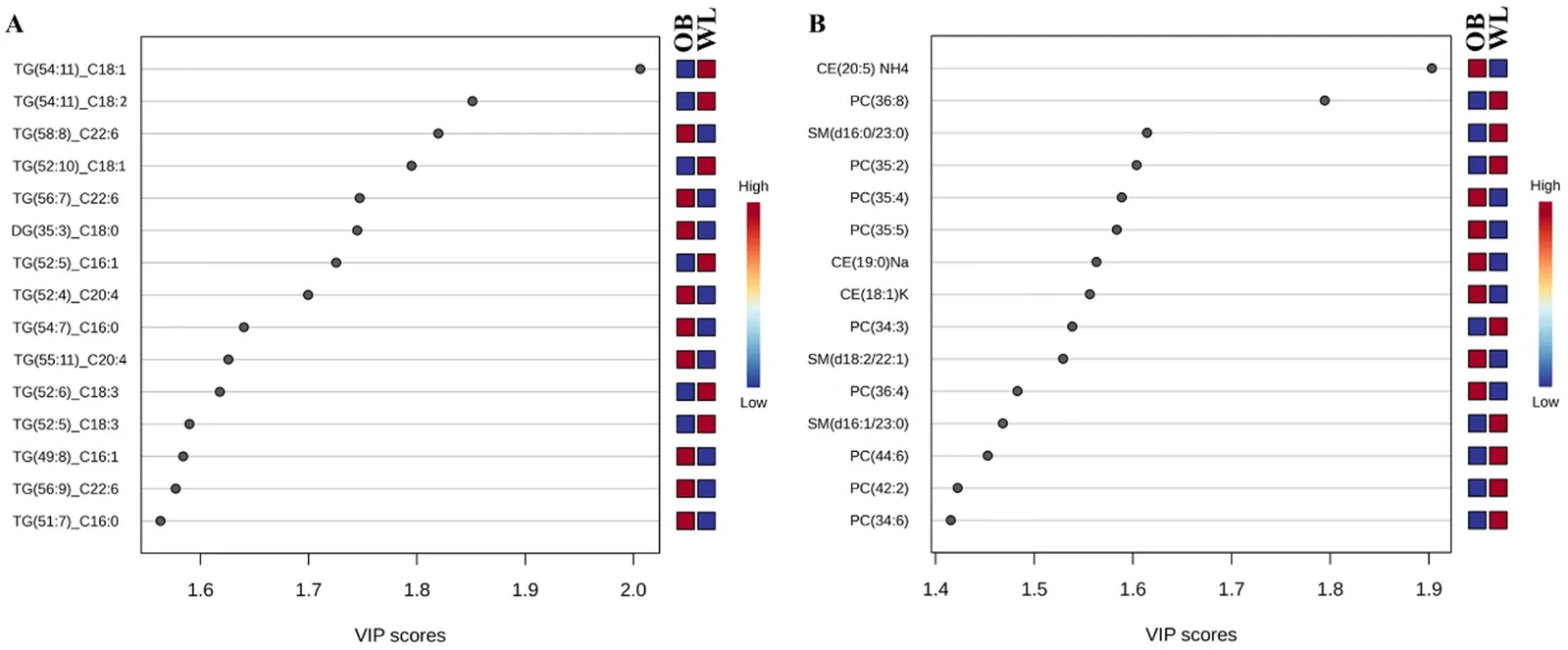
Variable importance in projection (VIP) plot of serum lipids that contributed a higher percentage of the explained residuals in the partial least squares-discriminant analysis plot in method 1 (A) and method 2 (B) in dogs with obesity before (OB) and after weight loss (WL).

Furthermore, PLS-DA (Figure 2) clearly separated the serum lipidomic profiles before and after weight loss, with high predictive ability for both method 1 (Figure 2A; Accuracy = 0.75; *Q*^2^ = 0.55; *R*^2^ = 1.0) and method 2 (Figure 2B; Accuracy = 0.88; *Q*^2^ = 0.63; *R*^2^ = 1.0). Based on VIP scores (Figure 3), the top 15 compounds that contributed a higher percentage of the explained residuals in the PLS-DA plot between OB and WL were highlighted for both method 1 and method 2.

After classifying lipids into their respective classes, dogs had higher serum concentrations of CE (*P* = 0.047), PC (*P* = 0.043), PI (*P* = 0.038), and SM (*P* = 0.023) before weight loss compared with after weight loss (Table 4). Total TG and DG concentrations, estimated from the lipidomic analysis, did not differ between OB and WL (*P* = 0.767 and *P* = 0.959, respectively; Table 4). However, following weight loss, specific TG and DG distribution categories differed.

**Table 4:** Serum lipid concentrations by lipid class in dogs with obesity before and after weight loss.

Lipid class, ng/µL	Before weight loss	After weight loss	SEM	*P*-value
**Diglyceride**	2.45	2.40	0.402	0.959
**Triglyceride**	1.27	1.15	0.190	0.767
**Lysophosphatidylethanolamine**	3.54	2.65	0.226	0.486
**Phosphatidylethanolamine**	0.052	0.048	0.0028	0.462
**Phosphatidylglycerol**	0.011	0.010	0.0005	0.373
**Ceramide**	0.0021	0.0019	0.0001	0.153
**Phosphatidylserine**	0.015	0.013	0.0006	0.084
**Lysophosphatidylcholine**	0.064	0.050	0.0039	0.076
**Cholesterol ester**	8.68	6.11	0.706	0.047
**Phosphatidylcholine**	0.0018	0.0017	0.0001	0.043
**Phosphatidylinositol**	0.125	0.087	0.0093	0.038
**Sphingomyelin**	1.10	0.79	0.072	0.023

SEM, standard error of the mean.

The relative abundance of 51-carbon TG was higher in OB (*P* = 0.003), whereas 52- and 54-carbon TG were higher in weight loss (*P* = 0.046 and *P* = 0.040, respectively; Table 5). Similarly, TG with 8 and 9 unsaturations were higher in OB (*P* = 0.009 and *P* = 0.005, respectively), whereas TG with 5 and 6 unsaturations were higher in WL (*P* = 0.042 and *P* = 0.044, respectively; Table 5). For DG, 37-carbon DG and DG with 6 unsaturations were higher in WL (*P* = 0.033 and *P* = 0.018, respectively; Table 6).

**Table 5:** Serum triglyceride profile according to carbon number and unsaturation degree in dogs with obesity before and after weight loss.

Trait	Before weight loss	After weight loss	SEM	*P*-value
**Carbon number**				
** 37**	0.26	0.30	0.038	0.619
** 39**	0.14	0.23	0.032	0.202
** 40**	0.07	0.09	0.012	0.385
** 41**	0.19	0.23	0.026	0.427
** 42**	0.35	0.43	0.051	0.439
** 43**	0.12	0.15	0.019	0.355
** 44**	0.76	0.97	0.112	0.366
** 45**	0.40	0.52	0.059	0.319
** 46**	1.11	1.44	0.152	0.302
** 47**	0.22	0.26	0.030	0.444
** 48**	1.57	1.81	0.146	0.431
** 49**	1.83	1.70	0.129	0.617
** 50**	3.83	4.14	0.137	0.266
** 51**	7.95	5.26	0.502	0.003
** 52**	11.26	13.62	0.603	0.046
** 53**	29.15	24.67	1.282	0.079
** 54**	12.10	14.86	0.688	0.040
** 55**	17.59	16.67	0.511	0.385
** 56**	6.90	8.17	0.537	0.254
** 57**	1.63	1.78	0.109	0.499
** 58**	2.24	2.33	0.200	0.826
** 59**	0.06	0.07	0.008	0.457
** 60**	0.29	0.31	0.035	0.735
**Unsaturation number**				
** 0**	1.30	1.73	0.185	0.264
** 1**	1.15	1.48	0.165	0.340
** 2**	1.50	1.71	0.147	0.490
** 3**	3.05	3.19	0.111	0.550
** 4**	8.59	9.98	0.460	0.135
** 5**	8.29	10.30	0.504	0.042
** 6**	5.37	6.67	0.329	0.044
** 7**	6.43	7.29	0.397	0.295
** 8**	10.82	8.60	0.459	0.009
** 9**	18.86	14.87	0.772	0.005
** 10**	23.61	22.18	0.683	0.314
** 11**	9.58	10.35	0.315	0.237
** 12**	1.26	1.43	0.092	0.378
** 13**	0.18	0.22	0.025	0.397
**Grouped unsaturation**				
** Monounsaturated**	2.65	3.19	0.311	0.404
** Polyunsaturated**	96.04	95.08	0.488	0.341

SEM, standard error of the mean.

**Table 6:** Serum diglyceride profile according to carbon number and unsaturation degree in dogs with obesity before and after weight loss.

Trait	Before weight loss	After weight loss	SEM	*P*-value
**Carbon number**				
** 28**	0.69	0.94	0.093	0.185
** 29**	0.10	0.09	0.007	0.483
** 30**	0.14	0.15	0.010	0.732
** 31**	0.09	0.09	0.007	0.689
** 32**	1.61	1.89	0.132	0.305
** 33**	0.66	0.64	0.046	0.807
** 34**	1.67	1.71	0.167	0.898
** 35**	2.07	2.91	0.246	0.085
** 36**	3.88	3.69	0.209	0.663
** 37**	1.46	1.86	0.097	0.033
** 38**	72.79	70.71	0.807	0.207
** 39**	12.53	12.72	0.247	0.708
** 40**	0.81	0.93	0.057	0.317
** 42**	0.66	0.80	0.048	0.146
** 43**	0.59	0.59	0.035	0.906
** 44**	0.24	0.27	0.019	0.463
**Unsaturation number**				
** 0**	2.30	2.45	0.103	0.499
** 1**	2.22	2.26	0.138	0.891
** 2**	75.38	73.41	0.649	0.135
** 3**	2.60	2.68	0.164	0.809
** 4**	0.20	0.19	0.026	0.855
** 5**	0.55	0.69	0.055	0.222
** 6**	2.77	3.90	0.250	0.018
** 7**	1.21	1.31	0.041	0.246
** 8**	11.84	11.98	0.230	0.772
** 9**	0.39	0.45	0.027	0.327
** 10**	0.55	0.69	0.044	0.101
**Grouped unsaturation**				
** Monounsaturated**	77.59	75.67	0.549	0.079
** Polyunsaturated**	20.10	21.88	0.505	0.077

SEM = standard error of the mean.

The LION enrichment analysis revealed several significantly enriched terms before and after weight loss (FDR ≤ 0.05; Table 7). The LION enrichment graph containing all enriched terms is provided in Figure S1.

**Table 7:** Significant (FDR ≤ 0.05) enriched terms in the Lipid Ontology (LION) enrichment analysis of the serum lipidome before and after weight loss in dogs with obesity.

LION term ID	Description	Annotated	FDR *q*-value
**LION: 0012010**	Membrane component	257	<0.001
**LION: 0000095**	Headgroup with positive charge/zwitter-ion	202	<0.001
**LION: 0000003**	Glycerophospholipids	184	<0.001
**LION: 0012080**	Endoplasmic reticulum	181	<0.001
**LION: 0080974**	Above average bilayer thickness	28	0.001
**LION: 0000465**	Neutral intrinsic curvature	121	0.001
**LION: 0000030**	Diacylphosphocholines	116	0.003
**LION: 0080981**	Below average lateral diffusion	22	0.005
**LION: 0012081**	Mitochondrion	36	0.005
**LION: 0000010**	Phosphocholines	134	0.008
**LION: 0002951**	Fatty acid with more than 24 carbons	6	0.009
**LION: 0080978**	Average lateral diffusion	25	0.012
**LION: 0080971**	High bilayer thickness	12	0.012
**LION: 0001740**	Above average transition temperature	48	0.015
**LION: 0022272**	C(25:0)	3	0.019
**LION: 0002966**	Fatty acid with less than 2 double bonds	69	0.019
**LION: 0080947**	Sphingosine	10	0.026
**LION: 0002926**	C(20:1)	4	0.026
**LION: 0000038**	Diacylphosphoethanolamines	26	0.026
**LION: 0080972**	Very high bilayer thickness	16	0.026
**LION: 0001741**	Below average transition temperature	82	0.033
**LION: 0080976**	Very low lateral diffusion	12	0.035
**LION: 0000011**	Phosphoethanolamines	30	0.035

Functional analysis was performed by preselecting cellular component, function, lipid classification, and physical or chemical properties as ontology sources in ranking mode. Significant terms are ranked by FDR *q*-value.

## Discussion

The development of targeted lipidomic techniques has significantly advanced our ability to investigate how obesity disrupts lipid metabolism and composition. Although substantial evidence has already been gathered in laboratory animals and humans (Pietiläinen et al. 2011; Mousa et al. 2019; Yin et al. 2021; Lin et al. 2023), studies in canine models remain scarce. To our knowledge, this is the first study to characterize the serum lipidome of dogs with obesity before and after a structured weight-loss program based on energy restriction. Therefore, the present findings should be interpreted primarily as lipidomic remodeling associated with weight loss, rather than as the isolated effect of obesity or as a comparison with healthy lean dogs in weight maintenance. Overall, weight loss was accompanied by marked changes in the serum lipidomic profile, suggesting that lipidomic analysis may capture metabolic adaptations to caloric restriction and body weight reduction that are not fully reflected by conventional lipid markers.

Prior to weight loss, dogs with obesity exhibited greater abundances of specific triglyceride species (TG(49:8), TG(51:7), TG(51:8), TG(52:4), TG(53:8), TG(54:7), TG(55:8), TG(55:9), TG(55:11), TG(56:7), TG(56:9), and TG(58:8)). Following weight loss, however, an increased abundance of three other TG species (TG(52:5), TG(52:6), and TG(54:11)) was observed. Although total TG concentration estimated from the lipidomic analysis did not differ before and after weight loss, specific TG distribution categories changed, particularly according to the number of unsaturations. Before weight loss, dogs showed higher relative abundance of TG with eight and nine unsaturations, whereas after weight loss, TG with five and six unsaturations were higher. These findings suggest that caloric restriction and weight reduction induced qualitative remodeling of the circulating TG profile rather than a uniform reduction in total TG concentration. This distinction is relevant because total lipid class concentrations may mask changes in individual lipid species or subclasses.

In humans, Stegemann et al. (2014) showed that species-level lipidomic analysis outperformed total lipid class concentrations in predicting cardiovascular disease risk. However, the lipid features associated with cardiovascular disease in that study, mainly shorter-chain and less unsaturated TG, differ from the pattern observed here. Therefore, this comparison should be interpreted as support for species-level lipid evaluation, rather than as evidence of a similar cardiovascular-risk phenotype in dogs. Similarly, Xenoulis et al. (2020) showed that dogs with naturally occurring pancreatitis had altered lipoprotein profiles despite normal serum triglyceride and cholesterol concentrations, reinforcing that conventional lipid markers may not fully reflect qualitative lipid remodeling.

Extending this concept, our findings reveal that the serum lipidome undergoes dynamic changes that are not captured by routine biochemical profiles. Even in the absence of clinical hyperlipidemia, dogs had higher concentrations of cholesterol esters before weight loss, along with differences in specific triglyceride species before and after weight loss. Together, these results reinforce the notion that qualitative aspects of the lipid profile may be more informative of metabolic status than total lipid concentrations alone. In dogs, similar limitations in conventional testing have been noted by Sieber-Ruckstuhl et al. (2022). The authors demonstrated that specific lipid signatures could distinguish between different endocrinopathies even when traditional lipid markers showed similar elevations. This suggests that clinical management should move towards evaluating lipid quality, such as the degree of saturation and chain length, rather than just quantity.

Class-based lipid concentrations revealed higher levels of CE, PC, PI, and SM in OB dogs compared to WL dogs. Following the concept of dynamic changes, we observed a highly specific behavior, although the total absolute concentration of PC decreased with weight loss, 13 specific PC species actually increased significantly in WL. This phenomenon suggests that weight loss promotes a metabolic remodeling of the lipidome rather than a simple depletion of fat stores. This reinforces the notion that the intraclass lipid species profile, in addition to the total class concentration, may be important for characterizing lipidomic remodeling associated with weight loss in dogs with obesity (Hornburg et al. 2023). The study by Forster et al. (2018) conducted a metabolomic analysis in 66 clinically healthy adult companion dogs classified as normal weight, overweight, or obese. Overall, dogs with obesity exhibited an increased concentration of serum PC. In the current study, specific PCs such as PC(34:3), PC(40:3), PC(40:4), and PC(42:2) showed increased concentrations after weight loss. These same PCs have also been linked to obesity in humans, as reported in the Human Metabolome Database (Wishart et al. 2022).

Although the relationship between glycerophospholipids and obesity is not yet fully understood, other studies have reported elevated glycerophospholipid concentrations in both dogs and humans with obesity (Godoy et al. 2015; Rauschert et al. 2016). In this study, five subclasses of glycerophospholipids were found to be significantly enriched, along with higher concentrations of PC and PI in dogs with obesity prior to weight loss. In addition, mitochondria and endoplasmic reticulum terms were also significantly enriched, which may indicate that the changes observed in circulating phospholipids are related to lipid metabolic pathways involving intracellular organelles. Since phospholipids are key components of mitochondrial and endoplasmic reticulum membranes, these findings may suggest alterations in phospholipid synthesis and membrane-associated lipid metabolism (Osman et al. 2011; Chen et al. 2023).

In humans, alterations in mitochondrial function have been associated with increased reactive oxygen species production and inflammatory signaling (Murphy 2013; Meikle and Summers 2017). However, this interpretation should be considered with caution, because the present study was based on serum lipidomics and did not directly evaluate tissue membrane composition, mitochondrial function, reactive oxygen species production, or inflammatory markers. Therefore, whether serum phospholipid remodeling after weight loss reflects changes in mitochondrial-related lipid metabolism and inflammatory responses remains a hypothesis to be addressed in future studies.

In addition to glycerophospholipids, other complex lipid classes such as sphingolipids have also garnered attention due to their involvement in cellular stress responses and inflammation. Sphingolipids represent a fundamental class of lipids, playing a crucial role in maintaining the homeostasis of cellular plasma membranes (Tani et al. 2007). Over the past decades, the metabolic pathways involved in the synthesis and degradation of sphingolipid-derived metabolites have been extensively investigated (Stunff et al. 2004). Sphingomyelins are among the most abundant components of the phospholipid membrane and give rise to several important bioactive metabolites, including ceramides, sphingosine, and sphingosine-1-phosphate (S1P) (Hannun et al. 2001; Saba and Hla 2004). The enzymatic catabolism of SM occurs through reversible interconversions among SM, ceramides, sphingosine, and S1P. In contrast, the degradation of S1P catalyzed by sphingosine-1-phosphate lyase is irreversible and leads to the formation of phosphoethanolamine, which can subsequently be converted into PE, and hexadecenal, which can be further metabolized into glycerophospholipids. Thus, this pathway can be summarized as sphingomyelin ⇌ ceramides ⇌ sphingosine ⇌ sphingosine-1-phosphate → phosphoethanolamine (Zhou and Saba 1998; Stunff et al. 2004; Tani et al. 2007; Slotte 2013). Therefore, the changes observed in SM, sphingosine, and specific PE species after weight loss may indicate remodeling of bioactive lipid pathways connecting sphingolipid and glycerophospholipid metabolism.

Dysfunctional sphingolipid metabolism is strongly associated with the development of various human diseases (Hanamatsu et al. 2014; Ögretmen 2018; Torretta et al. 2019). Ceramides, sphingosine, and S1P are bioactive lipids involved in key cellular signaling pathways and have been shown to contribute to insulin resistance and chronic inflammation in human obesity (Summers 2018; Chaurasia et al. 2021). Moreover, S1P has been widely linked to the pathogenesis of type 2 diabetes, cancer, and cardiorespiratory disorders (Maceyka et al. 2012; Guitton et al. 2020), and serum lipidomic studies in humans have associated sphingolipid species with obesity-related inflammation, cardiovascular disease, and cancer (Hornburg et al. 2023). Although no significant differences in ceramide concentrations were observed, before weight loss, dogs had higher levels of SM and a marked enrichment of sphingosine. The reduction in sphingomyelin levels following weight loss, together with increased concentrations of specific PE species, may reflect remodeling of sphingolipid-related pathways, potentially involving increased catabolism or turnover of SM and sphingosine. Because dogs in the present study had preclinical obesity, the sphingolipid changes observed after weight loss may indicate that serum lipidomics can detect lipid remodeling associated with weight reduction even in the absence of overt clinical conditions.

Beyond their metabolic implications in obesity, CE, PC, PE, PI, and SM are key structural components of cell membranes (Van der Veen et al. 2017). In the LION enrichment analysis, terms related to lipid physical properties and membrane organization were enriched, including neutral intrinsic curvature, transition temperature, lateral diffusion, and bilayer thickness. The enrichment of terms related to bilayer thickness is particularly noteworthy, as greater membrane compactness is associated with reduced cell membrane permeability (Frallicciardi et al. 2022). From a physicochemical perspective, bilayer thickness is influenced by phospholipid acyl-chain length, degree of unsaturation, and cholesterol incorporation, with longer acyl chains, lower unsaturation, and higher cholesterol content contributing to thicker bilayers (Gianfrancesco et al. 2018).

In this context, Weijers (2012) hypothesized that a shift from unsaturated to saturated phospholipid acyl chains in cell membranes may increase membrane thickness and reduce glucose transport via glucose transporter type 4 in humans. Similar mechanisms have been discussed in relation to insulin resistance in dogs with obesity (Weijers and Bekedam 2007; German et al. 2009). However, the present findings do not allow confirmation of this hypothesis, since the dogs in this study did not exhibit insulin resistance or clinical signs of metabolic syndrome (Tvarijonaviciute et al. 2012), and glucose transport, tissue membrane composition, membrane fluidity, and bilayer thickness were not directly evaluated. Therefore, the enriched LION terms related to bilayer thickness, lateral diffusion, transition temperature, and membrane organization should be interpreted as physicochemical annotations of the serum lipidome, rather than direct evidence of altered membrane structure. Nevertheless, these findings raise relevant hypotheses regarding the relationship between serum lipid remodeling after weight loss and membrane-associated lipid properties, which should be investigated in future studies.

Assigning biological significance to these results is a complex task, but essential to deepen our understanding of the role of lipids in modulating metabolic pathways in dogs with obesity and their implications for health. Canine obesity is commonly associated with hypertriglyceridemia and hypercholesterolemia, conditions that can worsen metabolic status or predispose animals to the development of other diseases (Jericó et al. 2009; Xenoulis and Steiner 2010). However, these abnormalities are not necessarily present in all dogs with obesity. In a review of canine hyperlipidemia, obesity was listed as a common secondary cause of hyperlipidemia, with hypertriglyceridemia and/or hypercholesterolemia reported in more than 25% of cases and ranging from mild to marked (Xenoulis and Steiner 2015). In this study, one of the inclusion criteria was the absence of hyperlipidemia, which limits the generalizability of the findings to the broader population of dogs with obesity, especially those with clinical obesity.

Additionally, although no differences were observed in total TG concentrations, the TG species profile between OB and WL exhibited marked shifts. These findings reinforce that meaningful lipid alterations can occur in cholesterol and triglycerides even in the absence of dyslipidemias, suggesting that the impact of obesity on these lipids involves more than just increased concentrations (Hornburg et al. 2023). However, the present study does not allow us to determine whether these lipid changes are a cause or consequence of obesity and its comorbidities; therefore, they should be interpreted as part of the lipidomic remodeling associated with weight loss. Nonetheless, the understanding that lipid metabolic disturbances extend beyond classical markers like cholesterol and triglycerides has become increasingly evident (Meikle and Summers 2017).

The present study demonstrates that weight loss following caloric restriction is accompanied by substantial serum lipidomic remodeling in dogs with obesity, even when conventional lipid markers remain within reference ranges. By characterizing changes in serum lipid species, classes, and pathways before and after a structured weight loss intervention, the present work reinforces the concept that obesity in dogs involves complex metabolic alterations that may be modulated by body weight reduction, rather than representing only a condition of excess body fat. The identification of cholesterol esters, phospholipids, and sphingolipids as key lipid classes altered after weight loss indicates that the lipidomic response extends beyond hypertriglyceridemia and hypercholesterolemia. From a clinical standpoint, these findings suggest that metabolically relevant lipid changes may occur even in dogs with preclinical obesity and routine lipid parameters within reference ranges, highlighting the limited sensitivity of traditional biochemical markers for assessing lipid metabolic responses to dietary intervention. These findings and their clinical significance are summarized in Box 1.

This study has limitations, including the relatively small sample size, which reflects the inherent challenges of conducting controlled nutritional and weight loss interventions in client-owned dogs. In addition, the inclusion of different breeds and body sizes, ranging from small to large dogs, may have contributed to interindividual variability and should be considered when interpreting the findings. The absence of a lean control group also limits direct comparisons between dogs with obesity and healthy dogs in weight maintenance, restricting causal inferences related specifically to obesity. Moreover, the post-weight loss condition should not be interpreted as equivalent to a healthy maintenance state, since calorie restriction and negative energy balance represent a distinct physiological condition. Nevertheless, the consistent lipidomic patterns observed before and after weight loss within the same individuals strengthen the biological relevance of the findings and provide a strong rationale for larger, controlled studies aimed at validating and expanding these observations.

**Box 1:** Translating canine lipidomics from molecular shifts to clinical practice.

**Key lipidomic findings in our study**	**Clinical significance**	**Possible physiological link**	**References**
**Shift in TG profile: OB dogs accumulated highly unsaturated TGs (8-9 unsaturations); WL dogs shift toward 5–6 unsaturations**	Total TG levels may appear normal, but the molecular profile reveals a high-risk metabolic state	Higher degrees of TG unsaturation are linked to increased susceptibility to lipid peroxidation and systemic inflammation	Xenoulis et al. (2020): Normal lipids mask pancreatitis risk. Sieber-Ruckstuhl et al. (2022): TG signatures distinguish endocrinopathies.
**Specific PC remodeling: 13 PC species (including PC 34:3, 40:4) increase after WL, despite a drop in total PC class concentration**	Monitoring specific PCs identifies “metabolic remodeling” and recovery of cellular homeostasis	Remodeling of PC species is crucial for maintaining membrane fluidity and anti-inflammatory signaling	Hornburg et al. (2023): Dynamic shifts in health/aging. HMDB (2022): Link to human pediatric obesity recovery.
**Bioactive sphingolipids: mild elevation of sphingomyelin in OB dogs and enrichment of sphingosine**	Detection of the “first signs” of endocrine and cardiac risk before clinical comorbidities emerge	Sphingolipids act as signaling for insulin resistance; mild shifts may signify a metabolism disturbance even in the absence of clinical manifestations	Mongraw-Chaffin et al. (2018): Metabolically healthy obesity may be transient and associated with later metabolic risk. Sieber-Ruckstuhl et al. (2022): Serum lipidomic signatures distinguished canine endocrinopathies beyond conventional biochemistry.

OB, dogs with obesity before weight loss; PC, phosphatidylcholines; TG, triglycerides; WL, dogs after weight loss.

## Supplementary Material

skag194_Supplementary_Data

## Data Availability

The data underlying this article will be shared on reasonable request to the corresponding author.

## Abbreviations

BCS: Body condition score
CE: Cholesterol ester
CAR: Acyl-carnitine
CER: Ceramide
DAL: Differentially abundant lipids
DG: Diglyceride
FDR: False discovery rate
FFA: Free fatty acid
LION: Lipid Ontology
MRM: Multiple reaction monitoring
MER: Maintenance energy requirement
MMS: Muscle mass score
OB: Dogs with obesity before weight loss
PC: Phosphatidylcholine
PE: Phosphatidylethanolamine
PG: Phosphatidylglycerol
PI: Phosphatidylinositol
PLS-DA: Partial least squares-discriminant analysis
PS: Phosphatidylserine
S1P: Sphingosine-1-phosphate
SM: Sphingomyelin
TG: Triglyceride
TW: Target weight
VIP: Variable importance in projection
WL: Dogs after weight loss
WLER: Weight-loss energy requirement
